# Bioavailability of Coenzyme Q_10_: An Overview of the Absorption Process and Subsequent Metabolism

**DOI:** 10.3390/antiox9050386

**Published:** 2020-05-05

**Authors:** David Mantle, Alex Dybring

**Affiliations:** 1 Pharma Nord (UK) Ltd., Telford Court, Morpeth, Northumberland NE61 2DB, UK; dmantle@pharmanord.co.uk; 2 Pharma Nord (DK) ApS, Sadelmagervej 30-32, DK-7100 Vejle, Denmark

**Keywords:** coenzyme Q10, bioavailability, ubiquinone, ubiquinol, absorption, transport, cellular metabolism

## Abstract

A lack of understanding of the processes determining the absorption and subsequent metabolism of coenzyme Q_10_ (CoQ_10_) has resulted in some manufacturers’ making incorrect claims regarding the bioavailability of their CoQ_10_ supplements, with potential consequences for the use of such products in clinical trials. The purpose of the present review article is, therefore, to describe the various stages of exogenous CoQ_10_ metabolism, from its first ingestion, stomach transit, absorption from the small intestine into the lymphatic system, transport in blood, and access into cells. In particular, the importance of CoQ_10_ crystal dispersion in the initial formulation is emphasised, the absence of which reduces bioavailability by 75%. In addition, evidence comparing the relative bioavailability and efficacy of ubiquinone and ubiquinol forms of CoQ_10_ has been reviewed.

## 1. Introduction

Coenzyme Q_10_ (CoQ_10_) is a substance similar to a vitamin in its biological functions, but by definition is not a vitamin because it can be synthesised by human cells. CoQ_10_ has an essential role supplying all cells with energy [1]. Specifically, CoQ_10_ shuttles electrons from complexes I and II to complex III of the mitochondrial respiratory chain. As such, it is an energy transfer molecule in the electron transport chain, which produces ATP chemical energy. CoQ_10_ has an important role as an antioxidant that protects lipid-soluble cell membranes [1]. In addition to its role in oxidative phosphorylation above, CoQ_10_ is a major lipid-soluble antioxidant, protecting cell membranes and circulating lipoproteins from oxidative damage by free radicals [1]. CoQ_10_ is the only lipid-soluble antioxidant manufactured within the body; in addition to preventing lipid peroxidation, CoQ_10_ is able to regenerate vitamins C and E back to their active fully reduced forms. The antioxidant function of CoQ_10_ is attributed to its reduced ubiquinol form, which must be constantly regenerated from its ubiquinone oxidised form. Moreover, a gene expression profiling study demonstrated that CoQ_10_ influences the expression of several hundred human genes [2]. In cell culture, CoQ_10_ has been shown to reduce the activity of inflammatory markers, suggesting that CoQ_10_ may have anti-inflammatory action via gene expression modification, most notably in elderly individuals [3,4].

Most of the body’s daily requirement for CoQ_10_ is produced within the body. Some CoQ_10_ is ingested from food (typically 5 mg/day [5]). It is estimated that the daily requirement for CoQ_10_, from both endogenous bio-synthesis and food sources, is about 500 mg; this estimate is based on a total body quantity of about 2 mg of CoQ_10_ and based on an average turnover time of 4 days in tissue [5]. This estimate serves as the justification for the size of the dosage (typically 300 mg/day) that is used in many clinical trial studies. 

As people mature into adulthood and begin to increase in age, the ability of the body to synthesise its own CoQ_10_ decreases; optimal human bio-synthesis occurs in the mid-twenties, with a continual gradual decline in tissue levels thereafter [6]. In addition to the normal aging process, CoQ_10_ levels have also been shown to be depleted in a number of disorders, particularly heart disease [7]. The synthesis of CoQ_10_ is a complex multistage process (governed by at least 13 genes), requiring a number of amino acids, vitamins, and trace element precursors and cofactors, deficiency of any of which can adversely affect normal CoQ_10_ production [8]. Nutritional supplementation with CoQ_10_ therefore provides a mechanism to maintain adequate levels within the body. 

A lack of understanding of the processes determining the bioavailability of CoQ_10_ has led some manufacturers to make incorrect claims relating to the bioavailability of their CoQ_10_ supplements; this in turn is of potential relevance to the use of such supplements in clinical studies. The purpose of this review article is, therefore, to describe the bioavailability process of supplemental CoQ_10_, from its first ingestion all the way through to its uptake and utilisation within the cells, in order to clarify these misleading marketing claims.

## 2. Initial Formulation

Although CoQ_10_ was originally purified from bovine heart tissue in the 1950s by Professor Frederick Crane [1], supplemental CoQ_10_ is now manufactured via a yeast fermentation method. The Kaneka corporation of Japan is now a major supplier of CoQ_10_ (produced via a fermentation process) to supplement manufacturers. The manufacturers then incorporate the CoQ_10_ into their respective products. CoQ_10_ produced via yeast fermentation produces the *trans*-form (as opposed to the *cis*-form) of CoQ_10_. This form is chemically identical to the form of CoQ_10_ that is bio-synthesised within the body. The CoQ_10_ raw material comes in the structural form of polymorphic crystals [9]. CoQ_10_ in polymorphic crystalline forms will not be absorbed in the gastro-intestinal tract. CoQ_10_ can be absorbed only as individual molecules, as described in Section 4 of this article below [10]. The CoQ_10_ crystals must therefore be dissociated first into individual CoQ_10_ molecules. These CoQ_10_ molecules should remain fully dissociated throughout the shelf life of the CoQ_10_ preparation. Not all CoQ_10_ manufacturers have demonstrated an ability to achieve this dissociation of the crystals to single molecules [10]. 

CoQ_10_ exists within the body in the oxidised form (ubiquinone) and the reduced form (ubiquinol); these two redox forms are continually inter-converted within cells as part of the normal function of CoQ_10_. In the blood, CoQ_10_ is transported in ubiquinol form (bound to low-density lipoprotein- and very low-density lipoprotein- cholesterol), irrespective of the initial dietary form (ubiquinone or ubiquinol) [11]. Because the initial absorption of CoQ_10_ into intestinal absorption cells was thought to be enhanced by the reduction of ubiquinone to ubiquinol [12], some still assume that ingestion of supplemental CoQ_10_ in the ubiquinol form will facilitate the absorption process. However, given that ubiquinol is, by its very nature, inherently unstable and will oxidise to ubiquinone, until relatively recently, CoQ_10_ supplements could only be produced using the ubiquinone form. Recent technical improvements in encapsulation methods have made the production of CoQ_10_ supplements in the ubiquinol form possible. On the basis of the above rationale, some manufacturers of reduced CoQ_10_ supplements have claimed that the reduced form of CoQ_10_ is more readily absorbed from the gastro-intestinal tract, even though recent absorption and bio-availability studies have brought the issue of ubiquinol’s claimed superior absorption into question, as explained in the sections that follow. 

Of particular note in this regard is the clinical study by Lopez-Lluch et al. [13]. The bioavailability of seven CoQ_10_ supplements differing in formulation (CoQ_10_ crystal dispersion status, type of carrier oil, composition of other excipients, and CoQ_10_ oxidation state) were administered in a single 100 mg dose to the same series of 14 healthy individuals, using a crossover/washout protocol. Bioavailability of the different formulations was quantified as the area under the curve (AUC) at 48 h. 

In CoQ_10_ studies, the AUC of the plasma CoQ_10_ concentration is used to report bioavailability because the AUC represents the proportion of the dose that has entered the blood circulation.

Cmax is the peak plasma CoQ_10_ concentration following the initial administration of a dose and prior to any subsequent administration.

The first point of note is the difference in bioavailability between samples 01 and 02 of the study. Both samples contained 100 mg CoQ_10_ in identical ubiquinone form, in a soy carrier oil with identical excipient content and capsule specification; sample 01 (Myoqinon, Pharma Nord, Vejle, Denmark) had been subject to a patented thermal crystal dispersion process whilst sample 02 (from the same manufacturer) had not undergone this procedure. The respective mean AUC and Cmax values in the Lopez-Lluch study were 28.0 mg/L/48 h and 1.07 mg/L for sample 01, and 6.89 mg/L/48 h and 0.33 mg/L for sample 2. Failure to subject crystalline CoQ_10_ to crystal dispersion therefore reduced bioavailability by approximately 75%. 

A second point relates to the relative bioavailability of the ubiquinone and ubiquinol forms of CoQ_10_. For the ubiquinol form of CoQ_10_ (sample 05), the AUC and Cmax values were 14.8 mg/L/48 h and 0.49 mg/L, respectively. Thus, the AUC for CoQ_10_ in ubiquinol form was approximately twice of that for ubiquinone, which had not been subjected to thermal crystal dispersion (sample 02), but was only 52% of that for ubiquinone that had been subjected to thermal crystal dispersion (sample 01). It should be noted that it is not possible to subject ubiquinol to the same crystal dispersion process as that described for sample 01; this is because of the differential stability of ubiquinone and ubiquinol. These data therefore demonstrate (i) the importance of CoQ_10_ crystal dispersion, the absence of which reduces bioavailability by some 75%; (ii) the fact that the relative bioavailability of ubiquinone and ubiquinol forms of CoQ_10_ depends on CoQ_10_ crystal dispersion status and carrier oil/excipient composition. 

Several previous clinical studies have reported no significant difference in the relative bioavailability of ubiquinone and ubiquinol supplements. Thus, a cross-over study by Vitetta et al. [14] in 11 healthy volunteers evaluated the bioavailability of 3 different CoQ_10_ formulations over a 6-week period, ubiquinone (150 mg), ubiquinol (150 mg), and a liposome ubiquinone formulation. There was a significant inter-subject variation in the baseline plasma CoQ_10_ levels, which persisted after administration of the test formulations. In addition, it was noted that the bioavailability of CoQ_10_ varied significantly between individuals, irrespective of the CoQ_10_ form (ubiquinone/ubiquinol) administered. There was no significant difference in plasma CoQ_10_ levels following administration of ubiquinone or ubiquinol formulations. The study therefore concluded that intestinal absorption of CoQ_10_ is highly variable among individuals, and is independent of the form of CoQ_10_ (ubiquinone/ubiquinol) administered. This is because the body is limited in how much CoQ_10_ it can absorb at a given time, and thus if an individual takes several 100 mg capsules together, some of the CoQ_10_ tends to be wasted [15]. 

Similarly in a 2002 study by Miles et al. [16], nine healthy adults were given single 180 mg doses of ubiquinone (LiQ-10 TM) or ubiquinol (Q-Nol TM) supplements, with a 2 week wash-out period; the results showed no significant difference between the two supplement types in raising plasma CoQ_10_ levels (determined as Cmax and AUC).

In contrast to the above, several studies have reported significantly improved bioavailability of ubiquinol versus ubiquinone supplements, particularly in older subjects. For example, a 2009 study by Evans et al. [17] supplemented 100 mg ubiquinone or ubiquinol as a single dose (with 2-week wash-out period) in 10 healthy adults aged over 60 years; the bioavailability (determined as Cmax and AUC) was significantly higher for the ubiquinol supplement. Similarly, a 2018 study by Zhang et al. [18] supplemented ubiquinone or ubiquinol (200 mg/day for 2 weeks, with 2-week wash-out period) in 10 older (>55 years) men; ubiquinol significantly increased plasma CoQ_10_ levels compared to ubiquinone but did not significantly increase ubiquinol levels compared to ubiquinone. In a 2014 study by Langsjoen and Langsjoen [19], supplementation with ubiquinone or ubiquinol (200 mg/day for 4 weeks with 4 weeks wash-out period) in 12 healthy adults resulted in significantly higher plasma CoQ_10_ levels with ubiquinol. Langsjoen did not report the increases in plasma ubiquinol levels. It should be noted that in all of these studies, the apparently superior bioavailabilty of ubiquinol was determined against supplemental ubiquinone samples that had not been subject to thermal dispersion, as described above.

A number of studies have been carried out with the objective of improving CoQ_10_ bioavailability using a variety of agents; examples include polyethylene glycol [20], phosphorylated tocopherols [21], polyoxamer/polyvinyl pyrrolidine [22], and hydrolysed proteins [23]. However, again the bioavailability of these formulations has not been compared directly with ubiquinone that has undergone crystal dispersion, the importance of which is demonstrated in the study by Lopez-Lluch et al. [13] above.

In summary, on the basis of the evidence reviewed above, the concept of the superior bioavailability of supplemental ubiquinol compared to ubiquinone would appear to be mistaken. This concept, widely disseminated on the Internet, seems in part to have originated from an inappropriate comparison between bioavailability data from studies by Shults et al. in 1998 [24] and Hosoe et al. in 2007 [25]. The older study involved the use of a dry powdered crystalline form of ubiquinone, which is poorly absorbed but was the only formulation available at the time. Thus, the comparison was not a head-to-head comparison in that it did not test the ubiquinol absorption results against a formulation of ubiquinone dissolved in appropriate carrier oils. The two studies being compared were conducted more than 10 years apart. They used different study subjects: healthy volunteers vs. patients with Parkinson’s disease, different investigators, different analytical labs, and different protocols.

## 3. Stomach Transit

CoQ_10_ is a lipid soluble substance; therefore, the highest quality CoQ_10_ preparations have the CoQ_10_ dissolved in a well-suited carrier lipid (e.g., soy oil or palm oil). Typically, they are encapsulated in bovine gelatine capsules. The capsule material does not seem to be an important factor for absorption. The gelatine capsules readily dissolve within minutes inside the stomach and release the oil-dissolved CoQ_10_. The stomach transit time varies among individuals and varies according to the type of food eaten. The complete transit of the CoQ_10_ from the stomach through the small intestines, and the lymph to the blood typically takes between 5 and 8 h [26]. The transited CoQ_10_ enters the duodenum as part of the chyme. Although the CoQ_10_ is in transit from the stomach into the duodenum, any CoQ_10_ in the reduced form, the ubiquinol form, will be oxidised to the ubiquinone form; in studies of CoQ_10_ absorption under conditions that simulate gastric conditions, this process usually takes about 90 min (Figure 20 in [26]). 

The lipid solubility of CoQ_10_ is a consequence of its inherent chemical structure—technically, it is due to the presence of an isoprenoid chain within the CoQ_10_ molecule. CoQ_10_ is not at all water soluble, and, contrary to some manufacturers’ claims, cannot be made water-soluble [26]. Thus, any alteration to the structure of the CoQ_10_ molecule in an attempt to increase water solubility means that the molecule is no longer CoQ_10_. Similarly, the presence of two additional hydrogen atoms within the chemical structure of ubiquinol (compared to ubiquinone) has a negligible effect on potential water solubility [26].

## 4. Duodenal Micellisation

In the duodenum, CoQ_10_ molecules are subject to the process of micellisation, the formation of mixed micelles, through which various lipid-soluble substances, such as monoglycerides, fatty acids, and fat-soluble vitamins, are prepared for intestinal absorption. Bile from the gall bladder is secreted into the duodenum. Substances present in the bile interact with CoQ_10_ molecules to form characteristic micellular spherical structures with a diameter of up to 20 nanometers. Each individual micelle may contain as many as thousands of CoQ_10_ molecules. Micelles are constantly breaking apart and then reforming. The micelles are small enough to diffuse between intestinal villi before they break apart. In this way, the micelles are transporting the CoQ_10_ molecules to the surface of the intestinal absorption cells preliminary to absorption. It is important to note that the micelles themselves are not absorbed; only single CoQ_10_ molecules that have been released from micelles can be absorbed by intestinal absorption cells [27].

## 5. Enterocyte Absorption of CoQ_10_ Molecules

The enterocyte cells that form the lining of the small intestines’ villi absorb CoQ_10_ via a process of “passive facilitated diffusion”. This process is described as passive because the process does not require energy. Carrier molecules facilitate the diffusion of the CoQ_10_ molecules through the cell membranes and into the enterocyte cells. Researchers have not definitively identified the carrier molecules for the CoQ_10_; however, some researchers have suggested the cholesterol transporter NPC1L1 (Niemann-Pick C1 Like 1) as a possible candidate [28,29]. 

In an attempt to understand CoQ_10_ absorption, Singh et al. [30] investigated the efficacy of various dosing strategies on serum CoQ_10_ levels in a group of 60 healthy adults. In the Singh study, a 200 mg CoQ_10_ dose yielded a larger increase in serum levels than a 100 mg dose. However, divided dosages of 2 × 100mg of CoQ_10_ yielded a larger increase in serum levels than a single 200 mg dose. The Singh data show that the human digestive system has a finite capacity to absorb CoQ_10_ in a single dose. This discovery of a limited capacity to absorb CoQ_10_ follows logically from the concept of the requirement for a CoQ_10_ transporter for access into enterocytes as outlined above.

Once inside the enterocytes, the CoQ_10_ molecules are incorporated into chylomicrons. Chylomicrons are lipoprotein-based particles that carry lipid-soluble substances and CoQ_10_ in the lymph and the blood circulation. From the basal surface of the enterocytes, chylomicrons are released by the active process of exocytosis. Chylomicrons are substances that are too large to enter the blood circulation via the process of capillary absorption within villi. For this reason, the chylomicrons transit into the lymph circulation within villi. The chylomicrons enter the distal abdominal lymph duct. The chylomicrons with their CoQ_10_ content then eventually enter the subclavian vein through the proximal abdominal and thoracic lymph ducts and continue in the blood circulation. 

## 6. Redox Conversion of CoQ_10_ Forms During Absorption

The CoQ_10_ molecules in the blood circulation are found predominantly in the reduced form. Accordingly, at some point, dietary CoQ_10_ in the oxidized ubiquinone form must therefore be reduced to the ubiquinol form; this occurs most probably in the lymph. Some CoQ_10_ supplement manufacturers are selling supplemental CoQ_10_ in the ubiquinol form because of the supposed superior bioavailability of the ubiquinol form of CoQ_10_. They are doing so because the absorption of CoQ_10_ into the enterocytes has been thought to require the reduction of ubiquinone to ubiquinol, an assumption that is based in part on work in which ubiquinone was reduced to ubiquinol during uptake by cultured Caco-2 human epithelial intestinal absorption cells [12]. Thus, the production of supplemental CoQ_10_ in the ubiquinol form was thought to facilitate the process of CoQ_10_ absorption, especially in patients with malabsorption disorders for whom the process of CoQ_10_ reduction process may be compromised, as well as in elderly individuals. 

## 7. CoQ_10_ Transport in the Blood

**Figure 1:**
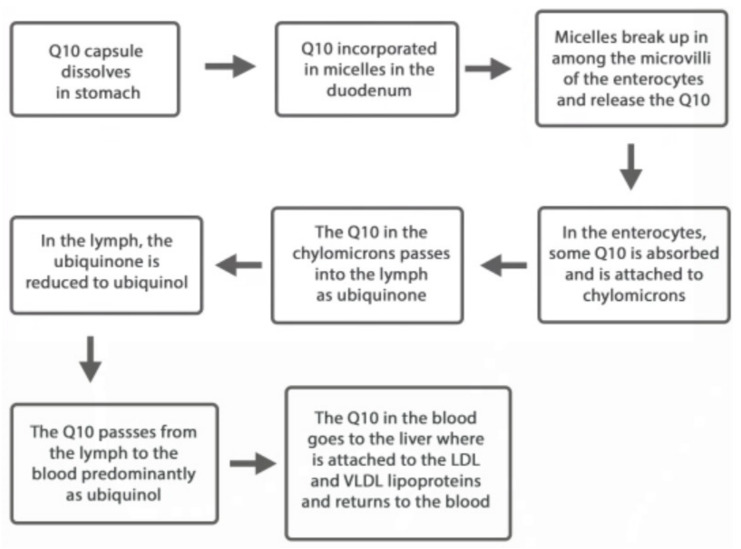
The transit of the ingested CoQ_10_ from the stomach to the blood circulation.

There is very little if any circulation of unbound CoQ_10_. The chylomicrons in the blood carry the CoQ_10_ to the liver, where it is primarily loaded into LDL and VLDL lipoprotein particles. A much smaller quantity of CoQ_10_ is packaged into high-density lipoprotein cholesterol. Moreover, the platelets and leucocytes in the blood contain CoQ_10_. The erythrocytes contain a quite small amount of CoQ_10_ (Figure 1).

The highest plasma concentration (Cmax) of the ingested CoQ_10_ is typically reached after about 6 hours. The half-life of the absorbed CoQ_10_ is about 33 h. Accordingly, the amount of time it takes for CoQ_10_ to reach a pharmacological steady state is rather prolonged (1-2 weeks). Normal plasma CoQ_10_ levels vary from person to person and are typically in the range 0.5 to 1.5 μg/mL [7]. Twice daily supplementation with 100 mg of CoQ_10_ has been reported to raise plasma CoQ_10_ levels from 0.90 to 3.25 mcg/mL, that is, to the level required for therapeutic efficacy in cardiovascular disease [7,31].

By inhibiting the oxidation of LDL cholesterol, CoQ_10_ seems to protect against atherosclerosis. There is a relatively low concentration of CoQ_10_ (0.5–0.8 mol/LDL particle) in LDL cholesterol compared to the content of *alpha* tocopherol (8–15 mol/LDL particle) in LDL cholesterol. Even so, CoQ_10_ has been shown to protect human low-density lipoprotein against lipid peroxidation better than alpha tocopherol [32]. The ratio of CoQ_10_ to LDL cholesterol has not been widely used as a bio-marker; however, there is evidence that the ratio of CoQ_10_ to LDL cholesterol may be more important in the prevention of atherosclerosis than is the HDL/LDL cholesterol ratio [33].

## 8. CoQ_10_ Utilisation Within Cells

CoQ_10_ is found in all cells. The relative tissue levels reflect the extent of metabolic activity and energy demand in various tissues and organs; accordingly, the highest CoQ_10_ levels are found in heart muscle tissue. Given its lipid nature, CoQ_10_ is assumed to enter the cell membranes by diffusion from the lipoprotein carriers in the blood; to date, there has been no known transporter of CoQ_10_ from blood into cells identified. Diffusion relies upon the movement of the CoQ_10_ molecules from areas of high CoQ_10_ concentration to areas of CoQ_10_ lower concentration; this need for a higher CoQ_10_ concentration in the blood than in the tissue cells is the rationale in clinical studies of heart failure patients for the use of CoQ_10_ dosages of 300 mg/day (and for plasma CoQ_10_ levels of at least 3 μg/mL). In this way, the movement of CoQ_10_ from the blood into tissues such as cardiac muscle will be facilitated [7]. 

There is some evidence that increased blood CoQ_10_ levels following supplementation is manifest as increased CoQ_10_ levels within tissues. Thus, a 2002 study by Rosenfeldt et al. in 24 older adults supplemented with 300 mg/day of CoQ_10_ or placebo for at least 7 days prior to cardiac surgery found that the CoQ_10_ level of atrial tissue was significantly increased in those taking CoQ_10_, especially in patients greater than 70 years of age [34]. In a 2008 study by Keith et al., patients with left ventricular dysfunction, supplementation with 150 mg/day of CoQ_10_ for 4 weeks before cardiac surgery increased CoQ_10_ concentrations in the heart but not in skeletal muscle [35]. In 2015, Okuyama et al. summarized the evidence that statin medications cause heart failure by depleting the heart muscle cells of Coenzyme Q_10_, heme A, and selenoproteins, thereby impairing mitochondrial ATP production [36]. 

Once within cells, several enzyme systems capable of maintaining CoQ_10_ in ubiquinol form have been identified. For example, cytochrome b5 reductase maintains CoQ10 in its reduced form [37]. Moreover, lipoamide dehydrogenase, glutathione reductase, and the seleno-enzyme thioredoxin reductase act in a multifunctional regeneration system to reduce ubiquinone to ubiquinol, thereby protecting cells from oxidative stress [38]. In addition, there is evidence that the enzyme NAD(P)H dehydrogenase quinone 1 (NQO1) acts as a component of the plasma membrane redox system. NQO1 enzymes act as a direct superoxide reductase to generate antioxidant forms of ubiquinone and vitamin E [39]. 

Recent studies by Bersuker et al. [40] and Doll et al. [41] have identified a membrane-associated oxidoreductase (FSP1) capable of reducing ubiquinone to ubiquinol during its passage through the membrane. Similarly, a study using human liver cancer cell line Hep G2 cells showed that intact cells could reduce the ubiquinone in low-density lipoproteins to ubiquinol, suggesting that a ubiquinone-reducing mechanism may exist in the plasma membrane, probably the outer surface, of Hep G2 cells. This mechanism may reduce extracellular ubiquinone to ubiquinol [42].

In animal models with Gram-magnitude dosages, CoQ_10_ has been shown to cross the blood–brain barrier; however, researchers have not been able to definitively show its ability to cross the blood–brain barrier in humans. The difficulty of getting supplemental CoQ_10_ into the brain with physiological dosages may explain the disappointing outcomes of some randomized controlled clinical trials supplementing CoQ_10_ in patients diagnosed with neurodegenerative disorders [24]. 

All cells except red blood cells have the capacity to manufacture CoQ_10_. CoQ_10_ researchers do not fully understand the relative contribution of cellular bio-synthesis of CoQ_10_ and dietary derived CoQ_10_ to cellular requirements. CoQ_10_ molecules are found primarily within the mitochondria (approximately 50% of total cellular CoQ_10_) but are also found in many other types of sub-cellular organelles such as the endoplasmic reticulum, the Golgi apparatus, lysosomes, and peroxisomes [43]. Regardless of the origin of CoQ_10_, whether endogenous or exogenous, a means of transport is required between these various sub-cellular organelles. This means of transport between and among organelles inside the cells is thought to involve a vesicle-mediated process that involves the Golgi apparatus. Although this transport process is not completely understood at present, it may involve saposin B as a presumptive binding and transfer protein for CoQ_10_ [44]. It should be noted that the important role of CoQ_10_ elsewhere within the cell also needs to be taken into account. 

## 9. Comparative Efficacy of supplemental Ubiquinone and Ubiquinol

Contrary to some manufacturers’ claims, ubiquinol is not the active form of CoQ_10_ compared to ubiquinone. Because ubiquinol and ubiquinone are continually inter-converted within the body, the concept that ubiquinol supplements may somehow be more efficacious than supplemental ubiquinone is incorrect, particularly in view of evidence reviewed above regarding relative bioavailability. However, it is correct that most randomised controlled clinical trials to date have been carried out using supplemental CoQ_10_ in ubiquinone form. This is particularly the case in relation to cardiovascular disease, where supplemental ubiquinone has been shown to be especially effective in two studies, Q-SYMBIO and KISEL-10. 

The Q-SYMBIO multi-national (nine country) study was carried out in 420 patients with chronic heart failure (NYHA class III and IV). The effect of CoQ_10_ supplementation (3 × 100 mg/day for 2 years) on symptoms and biomarker status (hence the trial acronym Q-SYMBIO) was assessed, as an adjuvant to conventional medication (angiotensin-converting enzyme (ACE) inhibitors, beta blockers). Assessment included clinical examination, echocardiography, and NT-pro-brain natriuretic peptide (NT-proBNP) status. The primary long-term endpoint was the time to first major adverse cardiovascular event (MACE), which included unplanned hospitalisation due to worsening heart failure and cardiovascular death. supplementation with CoQ_10_ significantly reduced the relative risk of MACE by 42%, with a reduction in both cardiac related deaths (43%) and all-cause mortality (42%). There was no significant difference in adverse events between the CoQ_10_-treated and placebo groups over the duration of the study [45]. Sub-group analysis of the effect of CoQ_10_ supplementation in the European cohort (231 of 420 patients) of the Q-SYMBIO study has since been undertaken [46]. In these patients, the relative risk of MACE was reduced by 67%, cardiac related mortality by 53%, and all-cause mortality by 55%; in addition, left ventricular ejection fraction was significantly improved by 6%, which was not observed in the original study. The improved outcome in the European cohort probably resulted from better patient compliance with supplementing CoQ_10_, resulting in a consistently higher plasma CoQ_10_ level for the duration of the study: 3.4 μg/mL at 3 months and 3.6 μg/mL at 2 years, compared to 3.0 μg/mL at 3 months and 2.1 μg/mL at 2 years in the full cohort. 

The KISEL-10 study was a clinical trial that enrolled 443 senior citizens living in south-eastern Sweden. The study was designed as a randomized, double-blind, placebo-controlled clinical trial. The elderly study participants—average age of 78 years—took either 200 milligrams of coenzyme Q_10_ together with 200 μg of an organic high-selenium yeast preparation or matching placebos daily for 4 years [47].

Professor Urban Alehagen and the team of researchers from Linköping University and Karolinska Institute reported significantly reduced risk of death from heart disease in the active treatment group. The relative risk reduction was 53%. The researchers also reported significantly improved heart function as shown on echocardiograms and significantly reduced levels of the N-terminal-proBNP bio-marker for heart muscle dysfunction in the active treatment group compared to the placebo group [47]. 

Moreover, the researchers quantified health-related quality of life outcomes. They used the Short Form-36, Cardiac Health Profile, and Overall Quality of Life survey questionnaires. The active treatment with a combination of CoQ_10_ and selenium was associated with a significant decrease in the number of days in the hospital and with significantly moderated deterioration in the senior citizens’ health-related quality of life [48].

Sub-study analysis of the data from the KiSel-10 study showed significant reductions in the blood levels of known bio-markers for inflammation—C-reactive protein and sP-selectin—and in known bio-markers for oxidative stress—copeptin and adrenomedullin [49,50].

The cardio-protective effect of the combined daily supplementation with CoQ_10_ and selenium endure for several years beyond the period of intervention. A follow-up analysis showed that the significantly reduced risk of death from heart disease still held 12 years after the intervention [51]. Professor Alehagen has identified the reductions in systemic inflammation, in oxidative stress, and in fibrosis of heart muscle tissue as possible mechanisms to explain the beneficial effect of combined coenzyme Q_10_ and selenium supplementation on the risk of heart disease in community living senior citizens [51,52].

There are unsubstantiated manufacturers’ claims circulating on the internet that individuals over 40 years of age may be progressively less able to absorb supplemental ubiquinone. However, the two studies above provide evidence that older individuals (Q-SYMBIO average age 63 years; KISEL-10 average age 78 years) are able to absorb and subsequently benefit from a properly formulated ubiquinone supplement (Table 1).

## 10. Conclusions 

As detailed above, the process of CoQ_10_ absorption is complex. It is not surprising that the absorption and bioavailability of CoQ_10_ supplements can vary widely and does indeed do so. This variability depends primarily on the formulation of the preparation. As there is considerable inter-individual variability in the uptake of CoQ_10_, the absorption and bioavailability of CoQ_10_ also depends on the capacity of a person to absorb a preparation with a given formulation. Thus, studies by both Wahlqvist et al. [53] and Lopez-Lluch et al. [13] demonstrate the ability of some individuals to absorb a particular formulation better than other formulation. The inter-individual variations in the absorption of CoQ_10_ were demonstrated as large inter-individual variations in the 36 h AUC and 48 h AUC, respectively, following the CoQ_10_ administration. 

With regard to the relative importance of ubiquinone and ubiquinol supplemental forms of CoQ_10_, there now seems to be evidence that there are multi-functional systems in place to convert ubiquinone to ubiquinol, involving as many as five different enzymes. Studies by Nordman et al. [38], Ross and Siegel [39], Bersuker et al. [40], Doll et al. [41], and Takahashi et al. [42] are of relevance. Takahashi et al. [42] established that isolated liver cancer cells are able to convert ubiquinone, either in the external medium or associated with LDL cholesterol, to ubiquinol, and that this conversion occurs due to the action of an outward-facing membrane-associated entity that was not further identified. The authors further consider that many other cell types may have the capacity to reduce ubiquinone to ubiquinol in the external cellular environment; if this is found to be the case, then presumably any of the various cell types lining the gastrointestinal tract would be able to facilitate this conversion, and the requirement for supplemental CoQ_10_ in reduced form to maximise absorption would be negated.

## Figures and Tables

**Table 1:** Clinical studies conducted with CoQ_10_ in the form of ubiquinone in individuals well over the age of 40 years.

Clinical Trial	Participants’ mean age	Outcome
Q-Symbio Study (2014)	62 years	Improved symptoms and survival in chronic heart failure patients
Kisel-10 Study (2013)	78 years	Improved heart function and reduced cardiovascular mortality in senior citizens
Italian Multi-Center Study (1993)	67 years	Fewer hospitalizations and fewer complications in chronic heart failure patients

## References

[1] Crane F.L. (2001). Biochemical functions of coenzyme Q_10_. J. Am. Coll. Nutr..

[2] Gutiérrez-Mariscal F.M., Yubero-Serrano E.M., Villalba J.M., Lopez-Miranda J. (2018). Coenzyme Q_10_: From bench to clinic in aging diseases, a translational review. Crit. Rev. Food Sci. Nutr..

[3] Schmelzer C., Lindner I., Rimbach G., Niklowitz P., Menke T., Döring F. (2008). Functions of coenzyme Q_10_ in inflammation and gene expression. BioFactors.

[4] Yubero-Serrano E.M., Gonzalez-Guardia L., Rangel-Zuñiga O.A., Delgado F.G., Gutiérrez-Mariscal F.M., Pérez-Martínez P., Delgado-Casado N., Cruz-Teno C., Tinahones F.J., Villalba J.M. (2011). Mediterranean Diet supplemented With Coenzyme Q10 Modifies the Expression of Proinflammatory and Endoplasmic Reticulum Stress-Related Genes in Elderly Men and Women. J. Gerontol. Ser. A Boil. Sci. Med Sci..

[5] Weber C., Bysted A., Hłlmer G. (1997). The coenzyme Q_10_ content of the average Danish diet. Int. J. Vitam. Nutr. Res..

[6] Kalen A., Appelkvist E., Dallner G. (1989). Age related changes in lipid composition of rat and human tissues. Lipids.

[7] Mantle D. (2015). Coenzyme Q_10_ and cardiovascular disease: An overview. Brit. J Cardiol..

[8] Mantle D. (2018). Hargreaves IP. Ataxia and coenzyme Q_10_: An overview. Brit. J. Neurosci. Nurs..

[9] Lee S.Q.E., Tan T.S., Kawamukai M., Chen E.S. (2017). Cellular factories for coenzyme Q_10_ production. Microb. Cell Factories.

[10] Moesgaard S., Paulin H.S. (2018). Recrystallization of Ubidecarenone for Improved Bioavailability. European Patent.

[11] Mohr D., Bowry V.W., Stocker R. (1992). Dietary supplementation with coenzyme Q_10_ results in increased levels of ubiquinol-10 within circulating lipoproteins and increased resistance of human low-density lipoprotein to the initiation of lipid peroxidation. Biochim. Biophys. Acta (BBA) Lipids Lipid Metab..

[12] Bhagavan H.N., Chopra R.K., Craft N.E., Chitchumroonchokchai C., Failla M.L. (2007). Assessment of coenzyme Q_10_ absorption using an in vitro digestion-Caco-2 cell model. Int. J. Pharm..

[13] López-Lluch G., Del Pozo-Cruz J., Sánchez-Cuesta A., Cortés-Rodríguez A.B., Navas P. (2019). Bioavailability of coenzyme Q_10_ supplements depends on carrier lipids and solubilization. Nutrition.

[14] Vitetta L., Leong A., Zhou J., Forno S.D., Hall S., Rutolo D. (2018). The Plasma Bioavailability of Coenzyme Q10 Absorbed from the Gut and the Oral Mucosa. J. Funct. Biomater..

[15] Singh R.B., Niaz M.A., Kumar A., Sindberg C.D., Moesgaard S., Littarru G.P. (2005). Effect on absorption and oxidative stress of different oral Coenzyme Q_10_ dosages and intake strategy in healthy men. BioFactors.

[16] Miles M.V., Horn P., Miles L., Tang P., Steele P., Degrauw T. (2002). Bioequivalence of coenzyme Q_10_ from over-the-counter supplements. Nutr. Res..

[17] Evans M., Baisley J., Barss S., Guthrie N. (2009). A randomized, double-blind trial on the bioavailability of two CoQ_10_ formulations. J. Funct. Foods.

[18] Zhang Y., Liu J., Chen X.Q., Oliver Chen C.Y. (2018). Ubiquinol is superior to ubiquinone to enhance Coenzyme Q_10_ status in older men. Food Funct..

[19] Langsjoen P.H., Langsjoen A.M. (2013). Comparison study of plasma coenzyme Q_10_ levels in healthy subjects supplemented with ubiquinol versus ubiquinone. Clin. Pharmacol. Drug Dev..

[20] Qin B., Liu L., Pan Y., Zhu Y., Wu X., Song S., Han G. (2017). PEGylated Solanesol for Oral Delivery of Coenzyme Q_10_. J. Agric. Food Chem..

[21] Pham A.C., Gavin P., Libinaki R., Ramirez G., Boyd B.J. (2017). A new lipid excipient, phosphorylated tocopherol mixture, TPM enhances the solubilisation and oral bioavailability of poorly water soluble CoQ_10_ in a lipid formulation. J. Control. Release.

[22] Choi J.S., Park J.-W., Park J.-S. (2019). Design of Coenzyme Q_10_ solid dispersion for improved solubilization and stability. Int. J. Pharm..

[23] Inada A., Oue T., Yamashita S., Yamasaki M., Oshima T., Matsuyama H. (2019). Development of highly water-dispersible complexes between coenzyme Q_10_ and protein hydrolysates. Eur. J. Pharm. Sci..

[24] Shults C.W., Beal M.F., Fontaine D., Nakano K., Haas R.H. (1998). Absorption, tolerability, and effects on mitochondrial activity of oral coenzyme Q_10_ in parkinsonian patients. Neurology.

[25] Hosoe K., Kitano M., Kishida H., Kubo H., Fujii K., Kitahara M. (2007). Study on safety and bioavailability of ubiquinol (Kaneka QH™) after single and 4-week multiple oral administration to healthy volunteers. Regul. Toxicol. Pharmacol..

[26] Judy W.V. (2018). Coenzyme Q_10_: An Insider’s Guide.

[27] Wang T.Y., Liu M., Portincasa P., Wang D.Q.-H. (2013). New insights into the molecular mechanism of intestinal fatty acid absorption. Eur. J. Clin. Investig..

[28] Sahoo S., Aurich M.K., Jonsson J.J., Thiele I. (2014). Membrane transporters in a human genome-scale metabolic knowledgebase and their implications for disease. Front. Physiol..

[29] Takekawa Y., Sato Y., Yamaki Y., Imai M., Noto K., Sumi M., Takekuma Y., Iseki K., Sugawara M. (2016). An Approach to Improve Intestinal Absorption of Poorly Absorbed Water-Insoluble Components via Niemann–Pick C1-Like 1. Boil. Pharm. Bull..

[30] Singh R.B., Wander G.S., Rastogi A., Shukla P.K., Mittal A., Sharma J.P., Mehrotra S.K., Kapoor R., Chopra R.K. (1998). Randomized, double-blind placebo-controlled trial of coenzyme Q_10_ in patients with acute myocardial infarction. Cardiovasc. Drugs Ther..

[31] Weis M., Mortensen S., Rassing M., Møller-Sonnergaard J., Poulsen G., Rasmussen S. (1994). Bioavailability of four oral Coenzyme Q_10_ formulations in healthy volunteers. Mol. Asp. Med..

[32] Stocker R., Bowry V., Frei B. (1991). Ubiquinol-10 protects human low density lipoprotein more efficiently against lipid peroxidation than does alpha-tocopherol. Proc. Natl. Acad. Sci. USA.

[33] Tomasetti M., Tomasetti M., Solenghi M.D., Littarru G.P. (1999). Distribution of antioxidants among blood components and lipoproteins: Significance of lipids/CoQ_10_ ratio as a possible marker of increased risk for atherosclerosis. BioFactors.

[34] Rosenfeldt F., Pepe S., Linnane A., Nagley P., Rowland M., Ou R., Marasco S., Lyon W. (2002). The effects of ageing on the response to cardiac surgery: Protective strategies for the ageing myocardium. Biogerontology.

[35] Keith M., Mazer C., Mikhail P., Jeejeebhoy F., Briet F., Errett L. (2008). Coenzyme Q_10_ in patients undergoing CABG: Effect of statins and nutritional supplementation. Nutr. Metab. Cardiovasc. Dis..

[36] Okuyama H., Langsjoen P.H., Hamazaki T., Ogushi Y., Hama R., Kobayashi T., Uchino H. (2015). Statins stimulate atherosclerosis and heart failure: Pharmacological mechanisms. Expert Rev. Clin. Pharmacol..

[37] Villalba J., Navarro F., Gómez-Díaz C., Arroyo A., Bello R., Navas P. (1997). Role of cytochrome b5 reductase on the antioxidant function of coenzyme Q in the plasma membrane. Mol. Asp. Med..

[38] Nordman T., Xia L., Björkhem-Bergman L., Damdimopoulos A., Nalvarte I., Arnér E.S.J., Spyrou G., Eriksson L.C., Björnstedt M., Olsson J.M. (2003). Regeneration of the antioxidant ubiquinol by lipoamide dehydrogenase, thioredoxin reductase and glutathione reductase. BioFactors.

[39] Ross D., Siegel D. (2017). Functions of NQO1 in Cellular Protection and CoQ_10_ Metabolism and its Potential Role as a Redox Sensitive Molecular Switch. Front. Physiol..

[40] Bersuker K., Hendricks J.M., Li Z., Magtanong L., Ford B., Tang P.H., Roberts M.A., Tong B., Maimone T.J., Zoncu R. (2019). The CoQ oxidoreductase FSP1 acts parallel to GPX4 to inhibit ferroptosis. Nature.

[41] Doll S., Freitas F.P., Shah R., Aldrovandi M., Da Silva M.C., Ingold I., Grocin A.G., Da Silva T.N.X., Panzilius E., Scheel C.H. (2019). FSP1 is a glutathione-independent ferroptosis suppressor. Nature.

[42] Takahashi T., Mine Y., Okamoto T. (2019). Extracellular coenzyme Q_10_ (CoQ_10_) is reduced to ubiquinol-10 by intact Hep G2 cells independent of intracellular CoQ_10_ reduction. Arch. Biochem. Biophys..

[43] Gane E.J., Weilert F., Orr D.W., Keogh G.F., Gibson M., Lockhart M.M., Frampton C.M., Taylor K.M., Smith R.A.J., Murphy M.P. (2010). The mitochondria-targeted anti-oxidant mitoquinone decreases liver damage in a phase II study of hepatitis C patients. Liver Int..

[44] Jin G., Kubo H., Kashiba M., Horinouchi R., Hasegawa M., Suzuki M., Sagawa T., Oizumi M., Fujisawa A., Tsukamoto H. (2008). Saposin B Is a Human Coenzyme Q10-Binding/Transfer Protein. J. Clin. Biochem. Nutr..

[45] Mortensen S.A., Rosenfeldt F., Kumar A., Dolliner P., Filipiak K.J., Pella D., Alehagen U., Steurer G., Littarru G.P. (2014). The Effect of Coenzyme Q_10_ on Morbidity and Mortality in Chronic Heart Failure. JACC Hear. Fail..

[46] Mortensen A.L., Rosenfeldt F., Filipiak K.J. (2019). Effect of coenzyme Q_10_ in Europeans with chronic heart failure: A sub-group analysis of the Q-SYMBIO randomized double-blind trial. Cardiol. J..

[47] Alehagen U., Johansson P., Björnstedt M., Rosén A., Dahlström U. (2013). Cardiovascular mortality and N-terminal-proBNP reduced after combined selenium and coenzyme Q_10_ supplementation: A 5-year prospective randomized double-blind placebo-controlled trial among elderly Swedish citizens. Int. J. Cardiol..

[48] Alehagan U., Aaseth J., Johansspon P. (2015). Reduced cardiovascular mortality 10 years after supplementaion with selenium and CoQ_10_ for 4 years: Follow up results of a randomised controlled trial in elderly citizens. PLoS ONE.

[49] Alehagen U., Lindahl T.L., Aaseth J., Svensson E., Johansson P. (2015). Levels of sP-selectin and hs-CRP Decrease with Dietary Intervention with Selenium and Coenzyme Q_10_ Combined: A Secondary Analysis of a Randomized Clinical Trial. PLoS ONE.

[50] Alehagen U., Aaseth J., Johansson P. (2015). Less increase of copeptin and MR-proADM due to intervention with selenium and coenzyme Q_10_ combined: Results from a 4-year prospective randomized double-blind placebo-controlled trial among elderly Swedish citizens. BioFactors.

[51] Alehagen U., Aaseth J., Alexander J., Johansson P. (2018). Still reduced cardiovascular mortality 12 years after supplementation with selenium and coenzyme Q_10_ for four years: A validation of previous 10-year follow-up results of a prospective randomized double-blind placebo-controlled trial in elderly. PLoS ONE.

[52] Alehagen U., Aaseth J., Alexander J., Svensson E., Johansson P., Larsson A. (2017). Less fibrosis in elderly subjects supplemented with selenium and coenzyme Q_10_-A mechanism behind reduced cardiovascular mortality?. BioFactors.

[53] Wahlqvist M.L., Wattanapenpaiboon N., Savige G.S. (1998). Bioavailability of two different formulations of CoQ_10_ in healthy subjects. Asia Pacific J. Clin. Nutr..

